# Menopause-induced uterine epithelium atrophy results from arachidonic acid/prostaglandin E2 axis inhibition-mediated autophagic cell death

**DOI:** 10.1038/srep31408

**Published:** 2016-08-10

**Authors:** Shengtao Zhou, Linjie Zhao, Tao Yi, Yuquan Wei, Xia Zhao

**Affiliations:** 1 Department of Gynecology and Obstetrics, Key Laboratory of Obstetrics & Gynecologic and Pediatric Diseases and Birth Defects of Ministry of Education, West China Second Hospital, and The State Key Laboratory of Biotherapy, West China Hospital, Sichuan University, Chengdu, 610041, P. R. China

## Abstract

Women experience menopause later in life. Menopause is characterized by dramatically decreased circulating estrogen level secondary to loss of ovarian function and atrophic state of genital organs. However, the molecular mechanisms for this process are not fully understood. In this study, we aimed to investigate the potential molecular mechanisms that underlie menopause-induced uterine endometrial atrophy. Our data showed that autophagy was activated in the uterine epithelial cells of both ovariectomized rats and peri-menopausal females. Endoplasmic reticulum (ER) stress occurred even prior to autophagy induction. Integrated bioinformatics analysis revealed that ER stress induced downstream decreased release of arachidonic acid (AA) and downregulation of AA/prostaglandin E2 (PGE2) axis, which led to Akt/mTOR signaling pathway inactivation. Consequently, autophagosomes were recruited and LC3-dependent autophagy was induced in uterine epithelial cells. Treatment with exogenous E2, PGE2, salubrinal or RNAi-mediated silencing of key autophagy genes could effectively counteract estrogen depletion-induced autophagy. Collectively, autophagy is a critical regulator of the uterine epithelium that accounts for endometrial atrophy after menopause.

Women experience menopause later in life. Menopause is characterized by dramatically decreased circulating estrogen level secondary to loss of ovarian function and atrophic state of genital organs. Previous studies demonstrated that the decline in ovarian function with menopause is associated with spontaneous increases in pro-inflammatory cytokines including IL-1, IL-6, and TNF-α. The accurate molecular mechanisms by which estrogen interferes with cytokine activity are still unelucidated but may potentially include interactions of the ER with other transcription factors, modulation of nitric oxide activity, antioxidative effects, plasma membrane actions, and alterations in immune cell function^1^. However, these molecular mechanisms could not explain the atrophic state of genital organs and tissues like uterine endometrium after menopause.

Autophagy, in particular macroautophagy, is a major ubiquitous catabolic process in eukaryotes facilitating the degradation of cytoplasmic components and organelles by selective or non-selective sequestration in double-membrane vesicles (termed autophagosomes)^2^. Although autophagy has been reported to be involved in a variety of pathological conditions including cancer, renal fibrosis, infectious diseases and autoimmune disorders, the functions of autophagy under normal physiological conditions are poorly annotated. In particular, while a previous study demonstrated that autophagy was implicated in the intricate control of human endometrial cycle^3^, the role of autophagy in postmenopausal endometrium still remains elusive.

In this study, we aimed to explore the potential molecular mechanisms responsible for estrogen withdrawal-induced uterine endometrium atrophy. As previous studies have demonstrated the important role of autophagy in cell size and tissue volume regulation upon estrogen depletion challenge^4^, we hypothesized that autophagy might participate in the uterine endometrial atrophy after menopause. Herein, we uncovered a critical role of autophagy as a regulator of the uterine endometrial atrophy after menopause in women.

## Materials and Methods

### Reagents and antibodies

Reagents used were as follows: letrozole (Sigma, L6545), 17β-estradiol (E2) (Sigma, 491187), celecoxib (Sigma, PZ0008), PGE2 (Sigma, P5640), tunicamycin (Sigma, T7765), salubrinal (Calbiochem, 324895), rapamycin (Sigma, R0395), arachidonic acid (Sigma, A9673), MTT (Sigma, M2128), Z-VAD-fmk (Sigma, V116), bafilomycin A1 (Sigma, B1793), 3-MA (Sigma, 08592), Hoechst 33342 (Sigma, B2261), acridine orange (Sigma, A6014), DMSO (Sigma, D2650). Letrozole, E2, celecoxib, PGE2, tunicamycin, and salubrinal were dissolved in DMSO, while MTT, Hoechst 33342, and acridine orange were dissolved in phosphate-buffered saline (PBS).

Antibodies were obtained from the following sources: antibodies against Beclin 1, COX-2, EP-4, β-actin, Bcl-2, BAX, phospho-4EBP1 and phospho-Akt (S473) were purchased from Santa Cruz Biotechnology (Santa Cruz, CA); Phospho-p70-S6K(T389), eIF2α, phospho-eIF2α, Raptor, phospho-mTOR(S2448), caspase 3, and phospho-S6(S235/236) were from Cell Signaling Inc (Beverly, MA); LC3, ATG5, protein disulfide isomerase (PDI) were from Abcam (Cambridge, MA); horseradish peroxidase (HRP)-conjugated anti-rabbit secondary antibody and horseradish peroxidase (HRP)-conjugated anti-mouse secondary antibody were purchased from Santa Cruz Biotechnology, Inc. (Santa Cruz, CA).

### Cell culture

Isolation of primary human uterine endometrial epithelial cells was performed as described previously^5^. Briefly, endometrium was obtained immediately after the uterus was removed, and was placed in an ice-cold 1:1 mixture of DMEM and Ham’s F-12 for transport to the laboratory. The tissue was vigorously pipetted to break up any remaining tissue pieces and passed over a stacked sterile wire sieve assembly with number 100 wire cloth sieve (140 μm size), followed by a number 400 wire cloth sieve (37 μm). After the endometrial digest was added to the top of the sieve assembly, the epithelial glands were retained in the number 100 and number 400 sieves while the stromal cells passed through to the container below. The glands were rinsed with a total of 50 ml of isolation medium before being back flushed out of the sieves onto a 100 mm sterile dish using the same medium. Any stromal cells remaining with the glands were further separated by selective adherence to plastic tissue culture dishes for 1 h. The clinicopathological data of patients from whom the samples for primary culture experiments were collected were listed in Supplementary Table 6. The primary human endometrial epithelial cells were cultured in media consisting of M199 and F12(1:1) with added Mitoplus^TM^ (2 ml/l) (Collaborative Biomedical Products), bovine pituitary extract (BPE; 2 ml/l) (Collaborative Biomedical Products), ITS+ (containing insulin (0.62 ng/ml), transferrin (0.62 μg/ml), and selenium (0.62 ng/ml), and antibiotic and antimycotic agents as described above.

Isolation of rat endometrial epithelial cells and rat endometrial stromal cells was conducted as described previously^6^,^7^. Briefly, rat endometrial epithelial cells and endometrial stromal cells were isolated from rat uterine horns. The uterine lumens were filled with PBS containing 0.1% collagenase (Worthington Biochemical Corporation, Lakewood, NJ) and incubated at 37 °C for 45 minutes in a shaking water bath. The dissociated cells, including both rat endometrial epithelial cells and endometrial stromal cells were washed with the basic culture medium Phenol red-free DMEM with Hams F-12, 1:1 (v/v) (DMEM/Hams F-12; Nacalai Tesque, Inc., Kyoto, Japan) containing 10% charcoal-stripped FBS (Hyclone Laboratories, Logan, UT, USA), and antibiotic and antimycotic agents as described above. Then, the cell suspension was plated onto 35 mm culture dishes, and allowed 1 hour of pre-incubation in a humidified atmosphere of 5% CO_2_ at 37 °C. After pre-incubation, non-attached rat endometrial endometrial epithelial cells and attached rat endometrial stormal cells were isolated for further functional validation.

### Animals and treatments

All animals were treated according to the guidelines for animal care approved by the Institutional Animal Care and Use Committee of Sichuan University (Chengdu, Sichuan, People’s Republic of China). Six-month-old female Sprague Dawley rats were used for our study. These animals were maintained in the experimental room at temperature 21–24 °C. After a 7-day adaptation period, bilateral ovariectomy or a sham operation was performed under pentobarbital sodium (30 mg/kg, i.p.) anesthesia. A longitudinal incision was made inferior to the rib cage on the dorsolateral body wall. The bilateral ovaries were exteriorized, ligated, and excised. Rats randomized to the sham surgery group had only a piece of fat excised. Five days after the surgery, rats were divided into seven groups: sham-operated group (sham, n = 12), OVX group (OVX, n = 12), OVX groups treated with E2 (10 μg/kg per day, s.c., n = 12), OVX groups treated with 3-MA (30 mg/kg, i.p., n = 12), OVX groups treated with Baf A1 (25 μg/kg, s.c., n = 12), OVX groups treated with celecoxib (25 mg/kg p.o., n = 12) and OVX groups treated with PGE2 (5 mg/kg, s.c., n = 12). At 1, 2, 4, and 6 weeks, three rats were sacrificed at each time point and the body weight and uterus weight were weighed. All experimental protocol were approved by Institutional Animal Care and Use Committee of Sichuan University.

### Transmission electron microscopy (TEM) analysis

Transmission electron microscopy analysis was done as described previously^8^. Briefly, cells were fixed in 0.1% glutaraldehyde in 0.1 M sodium cacodylate for 2 h, postfixed with 1% OsO_4_ for 1.5 h, washed and ultimately stained for 1 h in 3% aqueous uranyl acetate. The samples were then rinsed with water again, dehydrated with graded alcohol (50%, 75% and 95–100% alcohol) and embedded in Epon-Araldite resin (Canemco, 034). Ultrathin sections were cut on a Reichert Ultramicrotome, counterstained with 0.3% lead citrate and examined on a Philips EM420 transmission electron microscope. The cells with autophagic vacuoles were defined as cells that had 5 or more autophagic vacuoles. Values for the area occupied by autophagic vacuoles and the cytoplasm were obtained with Image Pro-Plus version 4.

### Detection of acidic vesicular organelles (AVOs)

Cells were placed on coverslips in 6-well plates and allowed to attach by overnight incubation. After treatment with DMSO (control), E2, letrozole or letrozole plus E2, cells were stained with 1 μg/mL acridine orange (AO) in PBS for 15 min, washed with PBS and examined under fluorescence microscope (Olympus Optical Co., Hamburg, Germany).

### GFP-LC3 transient transfection

Cells were transfected with pEGFP-LC3 plasmid using Lipofectamine 2000 reagent (Invitrogen, 11668027) according to the manufacturer’s instructions and were maintained on coverslips in 6-well plates. After transfection for 48 h, cells were treated with different reagents for 48 h. Subsequently, cells were washed with PBS twice and fixed by 4% paraformaldehyde for 15 min. The coverslides with cells were infiltrated with 60% glycerol on the microscope slides, and then the cellular localization pattern of GFP-LC3 protein was photographed using a fluorescence microscopy (Olympus Optical Co., Hamburg, Germany). The percentage of GFP-LC3-positive cells with GFP-LC3 punctate dots was caculated from 3 independent experiments. The cells with more than 5 GFP-LC3 punctate dots were counted under blinded conditions. A minimum of 100 total cells were counted at different random fields on each coverslide.

### siRNA transfection

siRNA oligonucleotides with specificity for Atg5 and Beclin 1 and respective nontargeting control siRNA consisting of a scrambled sequence were obtained from GenePharma. The specific siRNA oligonucleotide sequences were listed in Table S5. Cells were transfected with a total of 100 nmol/L of siRNA using LipofectAMINE 2000 (Invitrogen Life Technologies, Inc.) in antibiotic-free medium, according to the manufacturer’s instructions. Cells were harvested for functional study 48 hours after siRNA transfection with LipofectAMINE 2000.

### Bioinformatics analysis

For microarray analyses, we explored NCBI Gene Expression Omnibus (GEO) for datasets that might correlate ER stress with autophagy induction in the context of estrogen depletion, and three publicly available datasets (one analyzed Uterus treated by E2 in ovariectomized mice and two analyzed cells challenged with standard ER stress inducers) were used as summarized in Supplementary Table 1. The first, published by Hewitt *et al*., profiled the gene expression of uteri from ovariectomized C57BL/6 animals at various time points up to 24 hours following treatment with E2 and data were retrieved from GEO dataset GSE4664^9^. The second, published by Marciniak *et al*., analyzed fibroblast treated with tunicamycin to inducer ER stress and data were obtained from GEO dataset GSE2808^10^. The third dataset, published by Pulver-Kaste *et al*., analyzed cerebral vascular smooth muscle cells after treatment with thapsigargin and data were retrieved from GEO datasets GSE2883^11^. Venn diagram analysis was performed to identify significantly altered genes that are common after ovariectomy and ER stress challenge. Gene ontology (GO) enrichment was conducted by Onto-Tools^12^, which is a suite of tools for data mining based on information from GO. The identified genes involved in ER stress-induced autophagy in cells deficient in estrogen were clustered using Cluster 3.0 and Java Treeview^13^. The open source web-based tool STRING was utilized to analyze the protein-protein interaction networks as described previously^14^.

### PGE2 measurement

Both cell lysates and tissue samples were prepared and the PGE2 levels were determined using an enzyme-linked immunosorbent assay kit (Cayman Chemical) according to the manufacturer’s protocol.

### Arachidonic acid measurement

AA levels in human cell lysates and rat tissue lysates were measured using human AA ELISA kit and rat AA ELISA kis, respectively, from Cusabio Biotech Co., Ltd (Wuhan, China) following the manufacture’s protocol.

### Flow cytometry analysis

Cells were subjected to apoptotic analysis after each treatment for which the cells were harvested and washed with PBS and kept in 70% ethanol overnight at −20 °C. Then, the cells were centrifuged at 1,000 rpm for 5 min, suspended in PBS and treated with propidium iodide (PI) (1 mg/ml, Sigma Aldrich, St. Louis, Missouri). After an incubation period of 30 min in dark, the cells were analyzed using Becton Dickinson FACS caliber using ModFilt software on the acquisition of 10,000 cells for the final analysis.

### TUNEL assay

Samples were fixed, blocked, and permeabilized, and TUNEL was performed according to the manufacturers’ instructions.

### Immunoblotting

For immunoblotting, the whole-cell lysates and tissue lysates were prepared as described previously^15^. Briefly, proteins were extracted in RIPA buffer (50 mM Tris base, 1.0 mM EDTA, 150 mM NaCl, 0.1% SDS, 1% Triton X-100, 1% sodium deoxycholate, 1 mM PMSF) and quantified by the DC protein assay kit (Bio-Rad). Samples were separated by 12% SDS-PAGE and transferred to PVDF membranes (Amersham Biosciences). The membranes were blocked overnight with PBS containing 0.1% Tween 20 in 5% skimmed milk at 4 °C and subsequently probed by primary antibodies. Blots were incubated with respective primary antibodies for 2 h at room temperature. After washing three times in TBS with Tween 20, the blots were incubated with secondary antibody (diluted 1:10,000) conjugated to horseradish peroxidase for 2 h at room temperature. Blots were visualized by enhanced chemiluminescence reagents (Amersham Biosciences). β-actin was used as an internal control.

### Immunofluorescent microscropy

Immunofluorescent microscopy was carried out as described previously^16^. Secondary antibodies were donkey anti-rabbit Rhodamine Red-X (Jackson Immunoresearch Laboratories Inc.). Stained sections were viewed and photographed using a fluorescence microscope.

### Immunohistochemistry

We retrospectively included endometrial specimens from 15 menopausal women and 15 premenopausal women for pathological analysis who were subjected to surgery between 2009 and 2010 in West China Second Hospital, Sichuan University. This study was approved by the Institutional Ethics Committee of Sichuan University. Informed consents were obtained from all patients prior to analysis. For experiments related to human specimens, they were all carried out in accordance with the guidelines of the Institutional Ethics Committee of Sichuan University. Immunohistochemistry was carried out using the Dako EnVision Systems (Dako Cytomation GmbH, Hamburg, Germany) as described previously^17^. Briefly, consecutive paraffin wax-embedded tissue sections (3–5 μm) were dewaxed and rehydrated. Antigen retrieval was performed by pretreatment of the slides in citrate buffer (pH 6.0) in a microwave oven for 12 min. Thereafter slides were cooled to room temperature in deionized water. Endogenous peroxidase activity was quenched by incubating the slides in methanol containing 3% hydrogen peroxide followed by washing in PBS for 5 min after which the sections were incubated for 1 h at room temperature with normal goat serum and subsequently incubated at 4 °C overnight with the primary antibodies. Next the sections were rinsed with washing buffer (PBS with 0.1% bovine serum albumin) and incubated with horseradish peroxidase-linked goat anti-rabbit antibodies followed by reaction with diaminobenzidine and counterstaining with Mayer’s hematoxylin.

### Semiquantative RT-PCR

Total RNAs were isolated using Trizol reagent (Invitrogen) according to the manufacturer’s instructions. First-strand cDNA was reversely transcribed from 1 μg total RNA in a final volume of 20 μL using RTase and random hexamers from ExScript reagent kit (TAKARA, Dalian, China) according to manufacturer’s instructions. The primer sequences of autophagy genes and ER stress genes are listed in Tables S3 and S4, respectively. PCR reaction was performed as described previously^18^.

### Statistical analysis

Data are presented as mean ± SD of 3 independent experiments unless otherwise indicated. GraphPad Prism (GraphPad Software Inc., CA) was used for data analysis with all data assessed for normal distribution and equal variance. Based on staining intensities and positive rates, the immunoreactivity of each molecule was scored according to a previously reported semiquantitative scoring method^15^. Comparisons between two groups were performed Student’s *t* test and differences among multiple groups were evaluated by one-way ANOVA analysis. Differences were considered statistically significant at *P* < 0.05.

## Results

### Autophagy is activated in uterine endometrium in a murine ovariectomized model

**Figure 1:**
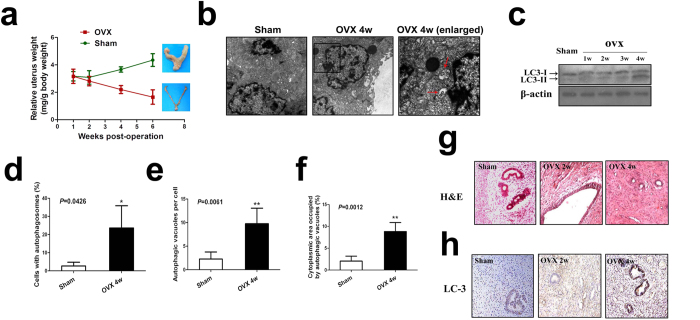
Estrogen depletion induces autophagy in uterine epithelium. (**a**) Relative uterus weight curves of female rats in sham-operated and OVX group. (**b**) TEM analysis of endometrium in sham and OVX group rats. An autophagosome is indicated by a red arrow. Black squares indicate the enlarged areas shown in insets. (**c**) Conversion of LC3-I (cytosolic) to LC3-II (autophagosome-bound) in ovariectomized rat uterine epithelium. (**d–f**) Quantification of percentage of cells with autophagosomes, autophagic vacuoles per cell and cytoplasmic area occupied by autophagic vacuoles from (**b**). (**g**) Rat uteri in sham and OVX group were analyzed by histology. (**h**) LC3 levels determined by immunohistochemistry in the uteri of sham and OVX group rats. All data are representative of three independent experiments. *p < 0.05; **p < 0.01; ***p < 0.001.

To examine whether autophagy plays a role in uterine endometrial atrophy of women after menopause, we used ovariectomized rats as a model to analyze the fate of uterine endometrium upon estrogen withdrawal. Our results showed that while ovariectomy significantly elevated body weights compared with those in the sham-operated group, it significantly reduced uterine weights as well as the ratio of uterine weight to body weight compared with those in sham-operated rats (2.13 fold, *P* < 0.05) (Fig. 1a). Transmission electron microscopy (TEM) analysis confirmed that an increased number of membrane-bound vacuoles characteristic of autophagosomes was observed in the cytoplasm of the uterine epithelial cells (not stromal cells) in ovariectomized rats 4 weeks postoperation whereas membrane-bound vacuoles could barely be found in the cytoplasm of the uterine epithelial cells in the sham-operated rats (Fig. 1b,d–f). Further immunoblotting analysis of the autophagy-associated protein LC3 indicated conversion from LC3-I to LC3-II, a defining feature of autophagy induction^19^, in uterine endometrium in a time-dependent manner after ovariectomy (Fig. 1c). Histological staining revealed that uterine endometrium became atrophic in a time-dependent manner after ovariectomy (Fig. 1g). More interestingly, immunohistochemical analysis further documented that accumulation of autophagosome occurred primarily in the glandular epithelial cells of uterine endometrium in ovariectomized rats, but we observed lower relative expression of LC-3 in the stromal cells (Fig. 1h). These findings demonstrated that autophagy might be activated in the uterine epithelium after estrogen withdrawal.

### Autophagy mediates estrogen withdrawal-induced uterine epithelial cell death

**Figure 2:**
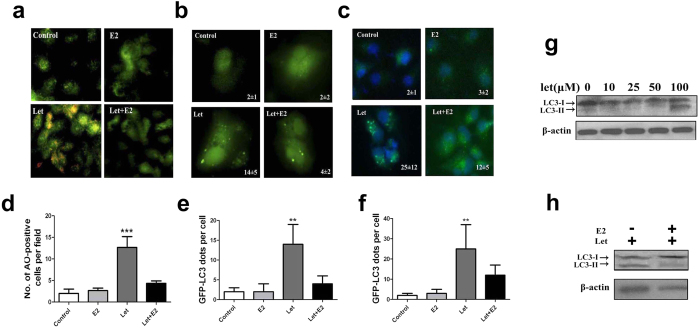
Autophagosomes are accumulated in estrogen-deprived EECs. (**a**) Acridine orange staining in EECs treated with DMSO (control), E2, letrozole or letrozole + E2 for 48 h. (**b**) EECs transfected with a GFP-LC3 plasmid were treated with DMSO (control), E2, letrozole or letrozole + E2 for 48 h and cells with GFP-LC3 punctate dots were examined at 48 h. (**c**) EECs treated with DMSO (control), E2, letrozole or letrozole + E2 for 48 h were analyzed for LC3 using immunofluorescent analysis. (**d**) Quantification of AO-positive autophagosomes in EECs treated with DMSO (control), E2, letrozole or letrozole + E2 from Fig. 1i. (**e**) Quantification of GFP-LC3 dots in EECs treated with DMSO (control), E2, letrozole or letrozole + E2 from Fig. 1j. (**f**) Quantification of LC3-positive dots in EECs treated with DMSO (control), E2, letrozole or letrozole + E2 from Fig. 1j. (**g**) Conversion of LC3-I to LC3-II in letrozole-treated EECs. (**h**) E2 rescues conversion of LC3-I to LC3-II in letrozole-treated EECs. All data are representative of three independent experiments. *p < 0.05; **p < 0.01; ***p < 0.001.

To further characterize the role of autophagy in endometrial cells after ablation of exogenous estrogen, we isolated primary endometrial epithelial cells (EECs) and primary endometrial stromal cells (ESCs) for further validation as described previously^3^. We examined the effects of letrozole, an established estrogen inhibitor, on both EECs and ESCs and found that estrogen depletion could trigger accumulation of acidic vesicular organelles (AVOs) in the cytosol of EECs (Fig. 2a) and the difference between the letrozole-treated group and other three groups was statistically significant (4.4 fold change, *P* < 0.001, Fig. 2d). In addition, pEGFP-LC3 plasmid was transiently transfected into EECs. As indicated in Fig. 2b, control cells demonstrated diffused and weak LC3 punctate dots, whereas letrozole-treated cells exhibited elevated number of green LC3 punctate dots in the cytosol, indicative of the recruitment of LC3-associated autophagosomes (Fig. 2b and 4.1 fold change, *P* < 0.01, Fig. 2e). Both the percentage of cells with GFP-LC3 dots and average number of GFP-LC3 dots per cell were increased. In addition, LC3 immunostaining also demonstrated elevated percentage of cells with LC3 dots and average number of LC3 dots per cell in EECs (Fig. 2c and 2.4 fold change, *P* < 0.01, Fig. 2f). However, we failed to observe any change in the number of green LC3 punctate dots in the cytosol of ESCs treated with either control or letrozole-treated cells (Supplementary Fig. 1). Dose-dependent LC3 conversion from LC3-I to LC3-II in EECs after letrozole treatment was confirmed by western blotting (Fig. 2g), which could be reversed by treatment of 17β-estradiol (E2) (Fig. 2h). These phenomena could be effectively offset by treatment of E2. Thus, estrogen ablation primarily triggers autophagosome formation in uterine epithelial cells.

**Figure 3:**
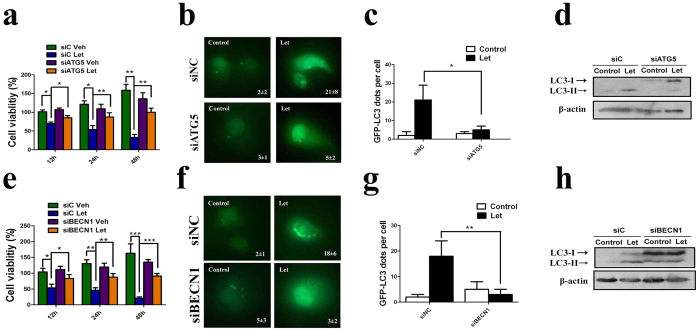
Silencing of autophagy genes inhibits letrozole-induced autophagy in uterine epithelial cells. (**a,b**) Effect of letrozole treatment and transfection with control siRNAs (siC) or ATG5-selective siRNAs (siATG5) on cell viability and LC-3 immunostaining. (**c**) Quantification of percentage of cells with LC3 dots transfected with siC or siATG5 prior to and after letrozole treatment relative to the total number of cells. (**d**) Effect of letrozole and transfection with siC or siATG5 on LC3 lipidation. (**e,f**) Effect of letrozole treatment and transfection with control siRNAs (siC) or Beclin 1-selective siRNAs (siBECN1) on cell viability and LC-3 immunostaining. (**g**) Quantification of percentage of cells with LC3 dots transfected with siC or siBECN1 prior to and after letrozole treatment relative to the total number of cells. (**h**) Effect of letrozole and transfection with siC or siBECN on LC3 lipidation. All data are representative of three independent experiments. *p < 0.05; **p < 0.01; ***p < 0.001.

Since autophagy has been implicated in either promotion or inhibition of cell survival^20^, we next investigated its participation in letrozole-mediated epithelial cell death induction. Silencing of autophagy-related gene Atg5 in EECs could strongly reduce letrozole-induced autophagy and cell death (Fig. 3a) and suppress letrozole-induced autophagosome formation (Fig. 3b–d). Similar results were observed in Beclin 1-silenced EECs (Fig. 3e–h). Taken together, these findings demonstrate that autophagy plays a prominent role in estrogen withdrawal-induced uterine epithelial cell death^21^.

### Estrogen withdrawal-induced autophagy promotes the apoptotic cell death of endometrial epithelium

**Figure 4:**
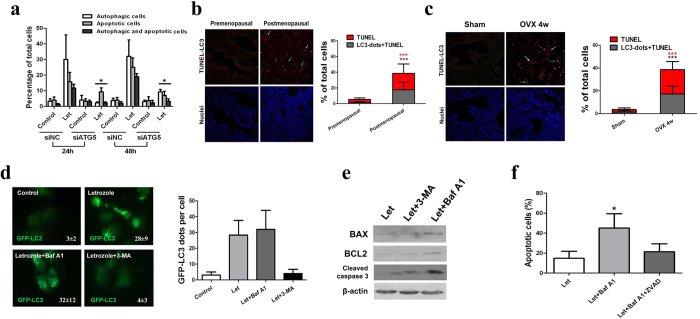
Estrogen depletion activates the autophagic cell death pathway *in vivo* and *in vitro*. (**a**) Effect of letrozole on autophagy and apoptosis of EECs transfected with siC or siATG5. White bars, cells with LC3 dots; grey bars, active caspase-3-positive cells; black bars, cells with both LC3 dots and active caspase-3 staining. Data correspond to the percentage of cells with LC3 dots (white bars), active caspase-3-positive cells (grey bars), and cells with LC3 dots and active caspse-3 staining (black bars) relative to the total number of transfected cells at each time point. (**b**) Left: LC3 immunostaining (red) and TUNEL (green) in premenopausal and postmenopausal endometrium. Arrows point to cells with LC3 dots and TUNEL-positive nuclei. Right: Bar graph shows the percentage of TUNEL-positive nuclei or cells with TUNEL-positive nuclei and LC3 dots relative to the total number of nuclei in each section. (**c**) Left: LC3 immunostaining (red) and TUNEL (green) in the endometrium of sham-operated and ovariectomized rats. Arrows point to cells with LC3 dots and TUNEL-positive nuclei. Right: Bar graph shows the percentage of TUNEL-positive nuclei or cells with TUNEL-positive nuclei and LC3 dots relative to the total number of nuclei in each section. (**d**) Left: EECs transfected with a GFP-LC3 plasmid were treated with DMSO (control), letrozole, letrozole + Baf A1 or letrozole + 3-MA and cells with GFP-LC3 punctate dots were examined at 24 h. Right: quantification of GFP-LC3 dots per cell in each group. (**e**) Western blot analysis of BAX, BCL2 and active caspase 3 protein levels in EECs treated with letrozole, letrozole + 3-MA or letrozole + Baf A1. (**f**) Quantification of percentage of apoptotic cells in EECs treated with letrozole, letrozole + Baf A1 or letrozole + ZVAD. All data are representative of three independent experiments. *p < 0.05; **p < 0.01; ***p < 0.001.

To delineate the role of autophagy in determination of uterine epithelial cell fate after estrogen depletion, we investigated whether letrozole-induced autophagy promoted the apoptotic death of EECs. Time-course analysis of LC3 immunostaining and TUNEL analysis in EECs revealed that autophagy preceded the appearance of apoptosis in EECs (Fig. 4a). Selective knockdown of ATG5 (Fig. 4a) prevented letrozole-induced apoptosis. We further evaluated the relationship between autophagy and apoptosis in both human uterine endometrium tissues and uterine endometrium of sham-operated and ovariectomized rats. We found that while premenopausal uterine epithelial cells demonstrated low level of autophagy and apoptosis, apoptosis was observed to be concomitant with autophagy in postmenopausal uterine epithelial cells (Fig. 4b). Similar conditions were also noticed in the uterine epithelial cells of ovariectomized rats compared with those in the sham group (Fig. 4c), indicating an apoptosis-inducing role of autophagy in estrogen-depleted uterine epithelium. We next characterized the role of autophagosome formation in apoptosis induction with 3-MA or Baf A1, which inhibits or promotes autophagosome accumulation, respectively^22^. LC3 punctate dots were significantly increased in the cytoplasm of letrozole-treated EECs compared with control and progressively accumulated in the cytoplasm after Baf A1 treatment. By contrast, in EECs treated with a combination of letrozole and 3-MA, LC3 punctate dots had a diffuse distribution throughout the cytoplasm (Fig. 4d). In this context, we examined cell death and apoptosis by measuring the expression levels of key apoptosis proteins and found that the expression levels of Bax, Bcl2 and cleaved caspase 3 proteins induced by letrozole did not change with 3-MA treatment. However, Baf A1 treatment remarkably increased Bax protein expression with no significant influence on Bcl2 protein expression. In addition, the expression level of cleaved caspase 3 protein in Baf A1-treated EECs was significantly higher than that in letrozole-treated or 3-MA-treated EECs (Fig. 4e). Moreover, cell apoptosis increased remarkably in EECs treated with Baf A1 compared to that in the letrozole-treated cells (Fig. 4f). The autophagosome accumulation-induced apoptotic cell death could be reversed by the caspase inhibitor Z-VAD-FMK (Fig. 4f). These observations suggest that the accumulation of autophagosomes could induce cell death in uterine epithelial cells.

To determine the relevance of autophagy and apoptosis in estrogen-withdrawn endometrium *in vivo*, we first examined whether inhibition of autophagy could reverse the activation of autophagy-mediated cell death in ovariectomized rats by measuring the change of ratio of uterus weight to body weight (relative uterus weight) in rats. Compared with sham-operated rats, the relative uterus weight was significantly decreased in ovariectomized rats, which could be reversed by treatment of E2. More interestingly, while the inhibition of autophagosome formation using 3-MA failed to decrease apoptosis or cell death manifested by increased relative uterus weight, the inhibition of autophagosome degradation by fusion with lysosomes using Baf A1 increased apoptosis and cell death and thus significantly decreased relative uterus weight, suggesting that the accumulation of autophagosomes induces apoptosis (Supplementary Fig. 2).

### Estrogen depletion initiates ER stress program prior to autophagy induction

**Figure 5:**
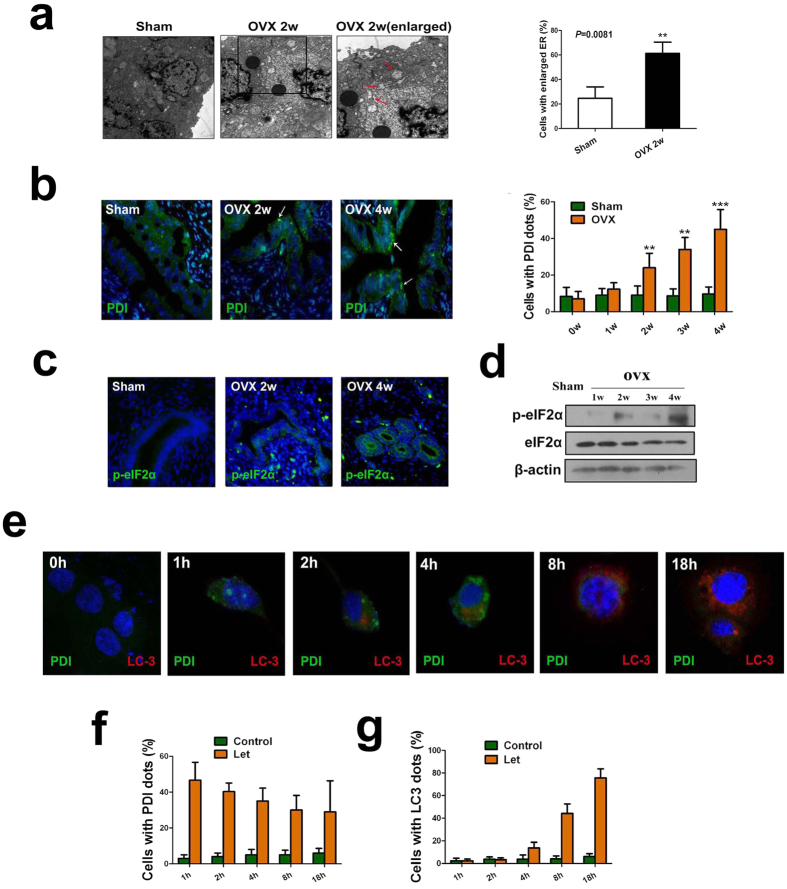
ER stress occurs prior to autophagy. (**a**) TEM analysis of endometrial epithelium in ovariectomized and sham-operated rats. Note the presence of the dilated ER in ovariectomized- but not sham-operated rat endometrium. Red arrows point to the ER. (**b**) Effect of ovariectomy on PDI immunostaining (green) in rat endometrial epithelium. (**c**) Effect of ovariectomy on p-eIF2α immunostaining (green) in rat endometrial epithelium. (**d**) Immunoblotting for p-eIF2α in endometrial epithelium of ovariectomized- and sham-operated rats. (**e**) Effect of letrozole on PDI (green) and LC3 (red) immunostaining in EECs at each time point. (**f**) Quantification of the percentage of EECs with PDI dots relative to total cell number at each time point after letrozole treatment. (**g**) Quantification of the percentage of EECs with LC3 dots relative to total cell number at each time point after letrozole treatment. All data are representative of three independent experiments. *p < 0.05; **p < 0.01; ***p < 0.001.

To characterize the early events that precedes autophagy in uterine epithelial cells after estrogen ablation, we utilized TEM to compare the ultramicroscopic structures of uterine epithelium in ovariectomized rats 2 weeks after operation with those in the sham-operated rats and found that the number of dilated endoplasmic reticulum (ER) in uterine epithelial cells increased significantly in ovariectomized rats 2 weeks after operation (Fig. 5a) as compared with the sham group, whereas the number of autophagosomes did not change obviously at that time point compared with the sham group. Immunostaining of the ER luminal marker protein disulphide isomerase (PDI) showed a remarkable dilation in the ER of uterine epithelial cells in ovariectomized rats (Fig. 5b), one of the defining features of ER stress. ER stress was also reported to be associated with increased phosphorylation of the α subunit of eukaryotic translation initiation factor 2 (eIF2α)^23^,^24^ and correspondingly a time-dependent increase in eIF2α phosphorylation in the uterine epithelium of ovariectomized rats was also observed (Fig. 5c).

*In vitro*, letrozole administration resulted in similar activation of ER stress prior to autophagy in EECs. TEM analysis showed that an accumulated number of dilated ER was observed whereas autophagosomes could not be detectable in EECs 1 h after letrozole treatment (Supplementary Fig. 3a,b). Time-course analysis of PDI and LC3 immunostaining in estrogen-depleted EECs demonstrated that enlarged ER (in green) could be observed as early as 1 h after letrozole treatment whereas LC-3-related autophagosomes (in red) increased 2 h after letrozole treatment (Fig. 5d). We next examined whether activation of ER stress occurred before the induction of autophagy in response to letrozole treatment in EECs on the molecular level. As shown in Supplementary Fig. 4a, letrozole treatment of EECs induced significant up-regulation of a series of ER stress genes including GADD45, GRP78, CHOP, TRIB3, and p8 after 1 hour incubation while only four autophagy genes (Atg5, LC3, Bnip3 and Beclin 1) were induced after 2 hours’ incubation with letrozole (Supplementary Fig. 4b). Collectively, these data indicated that ER stress occurs prior to autophagy in estrogen-depleted uterine epithelial cells.

### Estrogen depletion induces autophagy via ER stress-dependent AA/PGE2 axis inhibition

**Figure 6:**
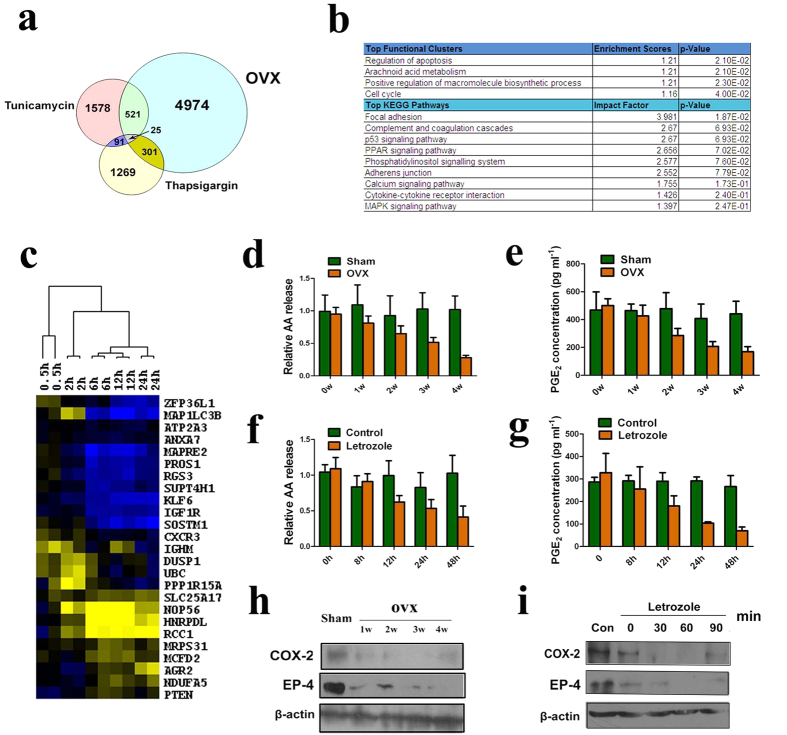
Critical role of AA/PGE2 axis in ovariectomy-triggered ER stress and autophagy. (**a**) Venn diagram that compares the gene expression profiles of three datasets reveal a set of 25 genes that are commonly altered under the conditions of tunicamycin treatment, ovariectomy and thapsigargin treatment. (**b**) Enrichment of functional clusters and pathways associated with ovariectomy-triggered ER stress and autophagy using the DAVID bioinformatics software. (**c**) Heatmap of expression patterns of the commonly altered 25 genes in the uteri from ovariectomized mice at various time points up to 24 hours following treatment with E2. (**d**) AA release in the uterus of ovariectomized- and sham-operated rats. (**e**) PGE2 concentration in the uterus of ovariectomized- and sham-operated rats. (**f**) AA release in letrozole-treated primary endometrial epithelial cells compared with control. (**g**) PGE2 concentration in letrozole-treated primary endometrial epithelial cells compared with control. (**h**) Western blot analyses of COX-2 and EP-4 expression in the endometrial epithelial cells of the uterus of ovariectomized- and sham-operated rats. (**i**) Western blot analyses of COX-2 and EP-4 expression in letrozole-treated primary endometrial epithelial cells compared with control. All data are representative of three independent experiments.

We next examined the potential signaling pathways that might correlate ER stress with autophagy induction in ovariectomized uterine endometrium by searching for genes commonly altered during ER stress and ovariectomy in Gene Expression Omnibus (GEO) datasets. Interestingly, we identified one dataset (GSE4664) that compared the global expression pattern of uterine RNA from ovariectomized mice treated with estradiol for various intervals with those of ovariectomized mice treated with control^9^. In addition, two datasets exploring the gene expression alterations prior to and after the treatment of tunicamycin and thapsingargin (two standard agents proved to induce ER stress)^10^,^11^, respectively, were also identified (shown in Supplementary Table 1). Gene expression analysis led to identification of 4974 genes, 1578 genes, and 1269 genes that were differentially expressed upon E2 treatment, tunicamycin treatment or thapsingargin treatment, respectively, in ovariectomized mouse uterine epithelium (*P* < 0.05). To further validate ER stress-induced genes that are involved in ovariectomy, we compared the gene expression profile in these three datasets and identified 25 genes that were commonly changed in these three datasets as illustrated by Venn diagram (Fig. 6a). The 25 genes have been shown in Fig. 6c and Supplementary Table 2. In particular, MAP1LC3B^25^ and SQSTM1^26^ are two important players in autophagic process, which were also found to be downregulated −2.51 fold and −17.4 fold in E2-treated ovariectomized mouse uterine epithelium compared with counterpart. These observations are in consistent with previous findings that estrogen could regulate the expression of MAP1LC3B^27^ and SQSTM1^28^. Moreover, STRING analysis revealed that MAP1LC3B and SQSTM1 constitute two important nodes in the protein-protein interaction network (Supplementary Fig. 5). This observation indicated that E2 could suppress ovariectomy-induced uterine epithelium autophagy, which indirectly proved that autophagic genes are activated in ovariectomized mouse uterine endometrium. Functional pathway analysis using DAVID bioinformatics software revealed that these genes participate in a variety of biological processes including apoptosis regulation, arachidonic acid (AA) metabolism, regulation of macromolecule biosynthetic process and cell cycle. The key KEGG pathways enriched in ovariectomy-induced ER stress include focal adhesion, complement and coagulation cascades, p53 signalling pathway, PPAR signaling pathway and MAPK signaling pathway (Fig. 6b).

Previous reports demonstrated that the expression of COX-2, as one of the key players in AA metabolism, could be reduced by ER stress^29^,^30^, we further examined the effects of estrogen depletion-induced ER stress on COX-2 expression and the activity of AA/prostaglandin E2 (PGE2) axis *in vivo* and *in vitro*. The concentrations of both AA and PGE2 in the uterine endometrium of ovariectomized rats were decreased in a time-dependent manner (Fig. 6d,e). *In vitro*, we explored the changes of AA and PGE2 concentrations in both letrozole-treated EECs and ESCs and found that AA and PGE2 concentrations were also reduced time-dependently in EECs (Fig. 6f,g). However, no significant changes of AA and PGE2 concentrations were found in letrozole-treated ESCs (data not shown). Moreover, western blot analysis of both endometrial epithelial cells of ovariectomized rat uterus and letrozole-treated human EECs revealed downregulation of COX-2 and prostaglandin E receptor 4 (EP4) (Fig. 6h,i), which indicated inhibition of AA/PGE2 signaling pathway after estrogen deprivation both *in vivo* and *in vitro*.

To explore whether this alteration is due to ER stress challenge, we analyzed letrozole-treated EECs and used tunicamycin-treated EECs as positive control through immunofluorescent analysis. Letrozole could downregulate the expression of COX-2 and EP-4 in EECs and similar results were also observed in tunicamycin-treated cells. These effects could be partially rescued by addition of E2 and salubrinal, an ER stress inhibitor, respectively (Supplementary Fig. 6a,b). In return, we found that increased level of PGE2 could decrease the level of ER stress while treatment of celecoxib significantly increased ER stress level (Supplementary Fig. 7). To further characterize the role of AA/PGE2 in autophagy induction in endometrial epithelial cells, we first transfected EECs with GFP-LC3 plasmid (EEC^GFP-LC3^ cells) and treated EEC^GFP-LC3^ cells with celecoxib, a selective COX-2 inhibitor, with tunicamycin used as positive control. Interestingly, the treatment of celecoxib induced an increasing number of green LC3 punctate dots in the cytosol of EECs. Both the percentage of cells with GFP-LC3 dots and average number of GFP-LC3 dots per cell were increased (Supplementary Fig. 8a,b). This effect could be inhibited by addition of PGE2 (Supplementary Fig. 8a,b). Likewise, GFP-LC3 dots were also increased in EEC^GFP-LC3^ cells treated with tunicamycin. This autophagic phenotype could be reversed by addition of salubrinal (Supplementary Fig. 8c,d).

*In vivo*, we further validated the critical role of AA/PGE2 axis in the uterus of ovariectomized rats and found that while treatment of PGE2 in ovariectomized mice did not significantly alter relative uterus weight, inhibition of COX-2 by treatment of celecoxib remarkably decreased relative uterus weight, indirectly implicated the involvement of AA/PGE2 axis in estrogen withdrawal-induced cell death in the uterus of ovariectomized rats (Supplementary Fig. 2). These observations demonstrated that estrogen depletion induces autophagy possibly *via* ER stress-dependent AA/PGE2 axis inhibition in uterine epithelial cells.

### Estrogen depletion-induced AA/PGE2 axis inhibition suppressed Akt-mTOR signaling pathway

**Figure 7:**
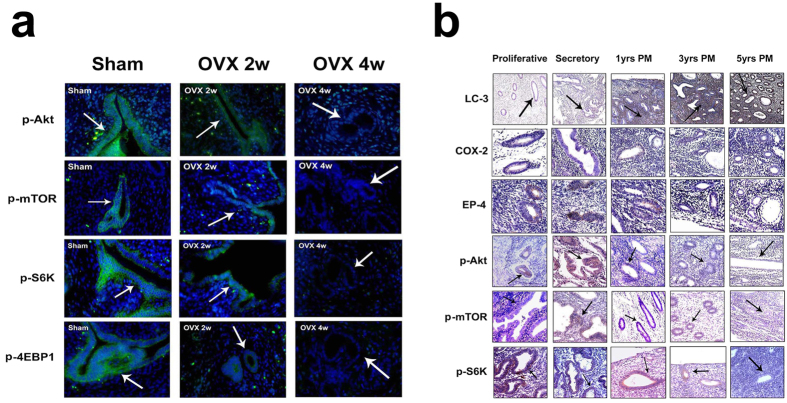
Akt-mTOR pathway is down-regulated in the endometrium of both ovariectomized rat uterus and postmenopausal females. (**a**) Effect of ovariectomy on p-Akt, p-mTOR, p-S6K and p-4EBP1 immunostaining (green) in rat endometrial epithelium. (**b**) Expression patterns of LC-3, COX-2, EP-4, p-Akt, p-mTOR, and p-S6K in premenopausal endometrium at either proliferative phase or secretory phase and in postmenopausal endometrium at indicated time points.

It is well established that inhibition of Akt-mTOR is a critical step in the early initiation of autophagy^31^. We next investigated whether estrogen depletion-induced AA/PGE2 inhibition could suppress Akt-mTOR pathway. *In vivo*, we examined whether ovariectomy could downregulate Akt-mTOR pathway. Immunohistochemistry analysis of human tissue specimens indicated that LC-3 expression increased primarily in human endometrial epithelium after menopause in an age-dependent manner (Fig. 7b), suggesting that human endometrial epithelium undergoes autophagic process after menopause. Previous reports indicated that mTORC1 activation could trigger phosphorylation of p70-S6K1 (T389), 4EBP1 (T37/46) and S6 (S235/235)^32^ whereas the activity of mTORC2 could be monitored by the mTORC2-directed phosphorylation of Akt at position Ser 473^33^. In our study, expression levels of COX-2 and EP-4 together with p-Akt (S473), p-mTOR (S2448), and p-p70-S6K1 (T389) in Akt-mTOR pathway were downregulated in an age-dependent manner in human endometrial epithelium (Fig. 7a). Similarly in rats, ovariectomy resulted in decreased phosphorylation of Akt (S473), mTOR (S2448), p70-S6K1 (T389) and 4EBP1(T37/46) in a time-dependent manner in rat uterus, as demonstrated by immunofluorescent analyses (Fig. 7a). This phenomenon indicated that estrogen deprivation could inactivate both mTORC1 and mTORC2. Hence, Akt-mTOR signaling pathway was suppressed in estrogen-depleted endometrial epithelium^34^,^35^.

## Discussion

**Figure 8:**
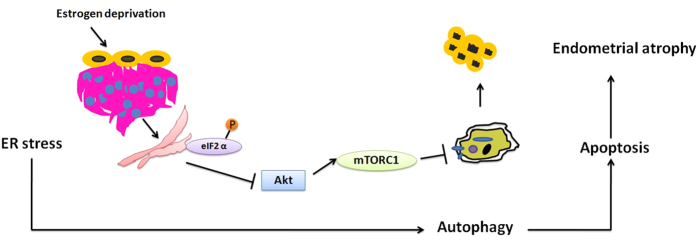
A schematic model summarizing the proposed mechanistic model leading to menopausal endometrial atrophy.

In this study, we demonstrated that autophagy was activated in the uterine epithelial cells of both ovariectomized rats and peri-menopausal females. Further studies proved that ER stress occurred even earlier than the induction of autophagy both *in vivo* and *in vitro*. Integrated bioinformatics analysis revealed that ER stress induced downstream downregulation of AA/PGE2 axis, triggering Akt/mTOR signaling pathway inactivation. Consequently, autophagosomes were recruited and LC3-dependent autophagy was induced in uterine epithelial cells. Treatment with exogenous E2, PGE2, salubrinal or RNAi-mediated silencing of key autophagy genes could effectively reverse estrogen depletion-induced autophagy. Collectively, autophagy is a critical regulator of the uterine epithelium that accounts for endometrial atrophy upon E2 withdrawal (Fig. 8).

Although the exact molecular mechanisms underlying the atrophic state of female genital organs after menopause and menopause-induced uterine endometrial atrophy are still far from clear, a previous study has demonstrated the involvement of autophagy and apoptosis in during the human endometrial cycle^3^. In particular, the autophagy-associated protein, microtubule-associated protein 1 light chain 3 alpha (MAP1LC3A, also termed LC-3) protein was mainly expressed within the endometrial glandular cells and its expression was increased during the secretory phase. The expression level of the membrane-bound form of LC3 (LC3-II) also increased as the menstrual cycle progressed, reaching a maximum level during the late secretory phase. This pattern coincided with the expression of cleaved caspase 3, indicating a close correlation between autophagy and apoptosis in the endometrium during menstrual cycle. Furthermore, expression of LC3-II and cleaved caspase 3 increased in the *in vitro*-cultured endometrial cancer cells when estrogen and/or progesterone were withdrawn from the culture media to simulate physiological hormonal changes. These findings lead us to hypothesize that autophagy is directly involved in the menopause-induced uterine endometrial atrophy and is correlated with apoptosis. Correspondingly, our experiments show that ovariectomy significantly reduced relative uterus weight compared with those in sham-operated rats. Histological staining revealed that uterine endometrium became atrophic in a time-dependent manner after ovariectomy. Further immunoblotting analysis demonstrated increased level of LC3-II in uterine endometrium after ovariectomy, indicating activation of autophagy in ovariectomized rat uterine endometrium. Immunohistochemical analysis further documented that accumulation of autophagosome occurred primarily in the glandular epithelial cells of uterine endometrium in ovariectomized rats, but rarely in the stromal cells. These observations were further validated in an *in vitro* model of estrogen withdrawn endometrium. Taken together, these results indicate that autophagy is activated in endometrial epithelium after estrogen withdrawal.

ER stress and autophagy are closely correlated under both physiological and pathological circumstances^36^,^37^,^38^. Although no direct evidence has linked ER stress with menopause-induced endometrial atrophy, Guzel *et al*. recently proved that the immunoreactivity of heat shock 70 kDa protein (HSPA5; also known as GRP78/BiP), a molecular chaperone within the endoplasmic reticulum and an well established ER stress molecule, in endometrial glandular and stromal cells was cycle-dependent, and was significantly higher in phases of the menstrual cycle when E2 levels are known to be the lowest compared with the rest of the cycle. *In vitro*, tunicamycin-induced HSPA5 expression was significantly lowered in these cells when pretreated with E2, indicating a cycle-dependent ER stress induction with a possible inverse correlation between ER stress and E2 levels in human endometrium^39^. Consistently, our data proved that ER stress also exists in the endometrium of both ovariectomized rats and menopausal females and ER stress occurred even earlier than autophagy. Using TEM analysis, we found that the number of dilated ER in uterine epithelial cells increased significantly in ovariectomized rats 2 weeks after ovariectomy, as compared with the sham group whereas the number of autophagosomes did not show apparent change at that time point compared with the sham group. Immunostaining of the ER luminal marker PDI also showed a remarkable dilation in the ER of uterine epithelial cells in ovariectomized rats. *In vitro*, letrozole administration resulted in similar activation of ER stress prior to autophagy in EECs. On the molecular level, letrozole treatment of EECs induced significant up-regulation of a series of ER stress genes including GADD45, GRP78, CHOP, TRIB3, and p8 after 1 hour incubation while only four autophagy genes (Atg5, LC3, Bnip3 and Beclin 1) were induced after 2 hours’ incubation with letrozole. Collectively, these data indicated that ER stress occurs prior to autophagy in estrogen-depleted uterine epithelial cells.

A previous study has reported the critical role of AA/PGE2 axis in endometrium^40^, which demonstrated that treatment of proliferative phase endometrium with PGE2 induced rapid phosphorylation of ERK1/2 proteins and a significant increase of cell proliferation in endometrial epithelial cells, indicating an important role of PGE2 in maintaining endometrial epithelial cell growth. In our study, an integrated analysis has unveiled the possible role of AA metabolism and in particularly, AA/PGE2 axis that might correlate ER stress with autophagy induction in ovariectomized uterine endometrium. Further functional studies revealed that the concentrations of both AA and PGE2 were decreased in both *in vivo* and *in vitro* estrogen-depleted model in a time-dependent manner. Moreover, immunoblotting analysis of both ovariectomized rat uterus and letrozole-treated EECs revealed downregulation of COX-2 and EP4, lending further support to the notion that AA/PGE2 signaling pathway was inhibited after estrogen deprivation. Down-regulated AA/PGE2 axis further suppressed akt/mTOR signaling pathway to induce autophagy in estrogen-withdrawn endometrium.

Autophagy either functions as a pro-survival mechansim or triggers cell death cascade activation^22^. As for the pro-death function of autophagy, the induction of apoptosis was promoted by the accumulation of autophagosomes in the cytoplasm. Our findings suggest that autophagy may induce apoptotic cell death in estrogen-withdrawal endometrium. 3-MA inhibits the formation of autophagosomes by inhibiting the activity of class III phosophoinositide-3 kinases, which are involved in the conversion of LC3-I to LC3-II. In contrast, Baf A1 causes autophagosome accumulation by reducing the removal of autophagosomes through fusion with lysosomes, producing a cellular morphology initially consistent with autophagy. Correspondingly, we observed that LC3 punctate dots were significantly increased in the cytoplasm of letrozole-treated EECs compared with control and progressively accumulated in the cytoplasm after Baf A1 treatment. By contrast, in EECs treated with a combination of letrozole and 3-MA, LC3 punctate dots had a diffuse distribution throughout the cytoplasm. Further functional studies demonstrated that cell apoptosis was increased remarkably in EECs treated with Baf A1 compared to that in the letrozole-treated cells. In addition, the expression levels of Bax, Bcl2 and cleaved caspase 3 proteins induced by letrozole did not change with 3-MA treatment. Moreover, the expression level of cleaved caspase 3 protein in Baf A1-treated EECs was significantly higher than that in letrozole-treated or 3-MA-treated EECs. The autophagosome accumulation-induced apoptotic cell death could be reversed by the caspase inhibitor Z-VAD-FMK. *In vivo*, we observed that while the inhibition of autophagosome formation using 3-MA failed to decrease apoptosis or cell death manifested by increased relative uterus weight, the inhibition of autophagosome degradation by fusion with lysosomes using Baf A1 increased apoptosis and cell death and thus significantly decreased relative uterus weight. These data implicated that estrogen withdrawal could promote autophagic cell death in uterine endometrium.

Collectively, our data show that ER stress is induced shortly after ovariectomy, which further leads to AA/PGE2 axis inhibition and Akt/mTOR pathway inhibition. These molecular events ultimately result in the recruitment of autophagosomes and induction of LC3-dependent autophagy in the uterine epithelium. Treatment with exogenous E2, PGE2, salubrinal or siRNA of key autophagy genes could effectively counteract estrogen depletion-induced autophagy in endometrial epithelial cells. Thus, our study has provided new clues to the molecular mechanisms of atrophy of postmenopausal endometrium.

## Additional Information

**How to cite this article**: Zhou, S. *et al*. Menopause-induced uterine epithelium atrophy results from arachidonic acid/prostaglandin E2 axis inhibition-mediated autophagic cell death. *Sci. Rep.* **6**, 31408; doi: 10.1038/srep31408 (2016).

## Supplementary Material

Supplementary Information
