# Author Correction: Menopause-induced uterine epithelium atrophy results from arachidonic acid/prostaglandin E2 axis inhibition-mediated autophagic cell death

**DOI:** 10.1038/s41598-020-63752-8

**Published:** 2020-04-20

**Authors:** Shengtao Zhou, Linjie Zhao, Tao Yi, Yuquan Wei, Xia Zhao

**Affiliations:** Department of Gynecology and Obstetrics, Key Laboratory of Obstetrics & Gynecologic and Pediatric Diseases and Birth Defects of Ministry of Education, West China Second Hospital, and The State Key Laboratory of Biotherapy, West China Hospital, Sichuan University, Chengdu, 610041 P. R. China

Correction to: *Scientific Reports* 10.1038/srep31408, published online 10 August 2016

This Article contains an error in Supplementary Figure 6b introduced during the re-editing of figures at re-submission, where the image for the Celecoxib treatment group was accidentally misplaced and is a duplication of the image for the Tunicamycin treatment group. This change does not affect the results or conclusions reported in the Article. The correct Supplementary Figure 6b is provided below as Figure 1.

**Figure 1:**
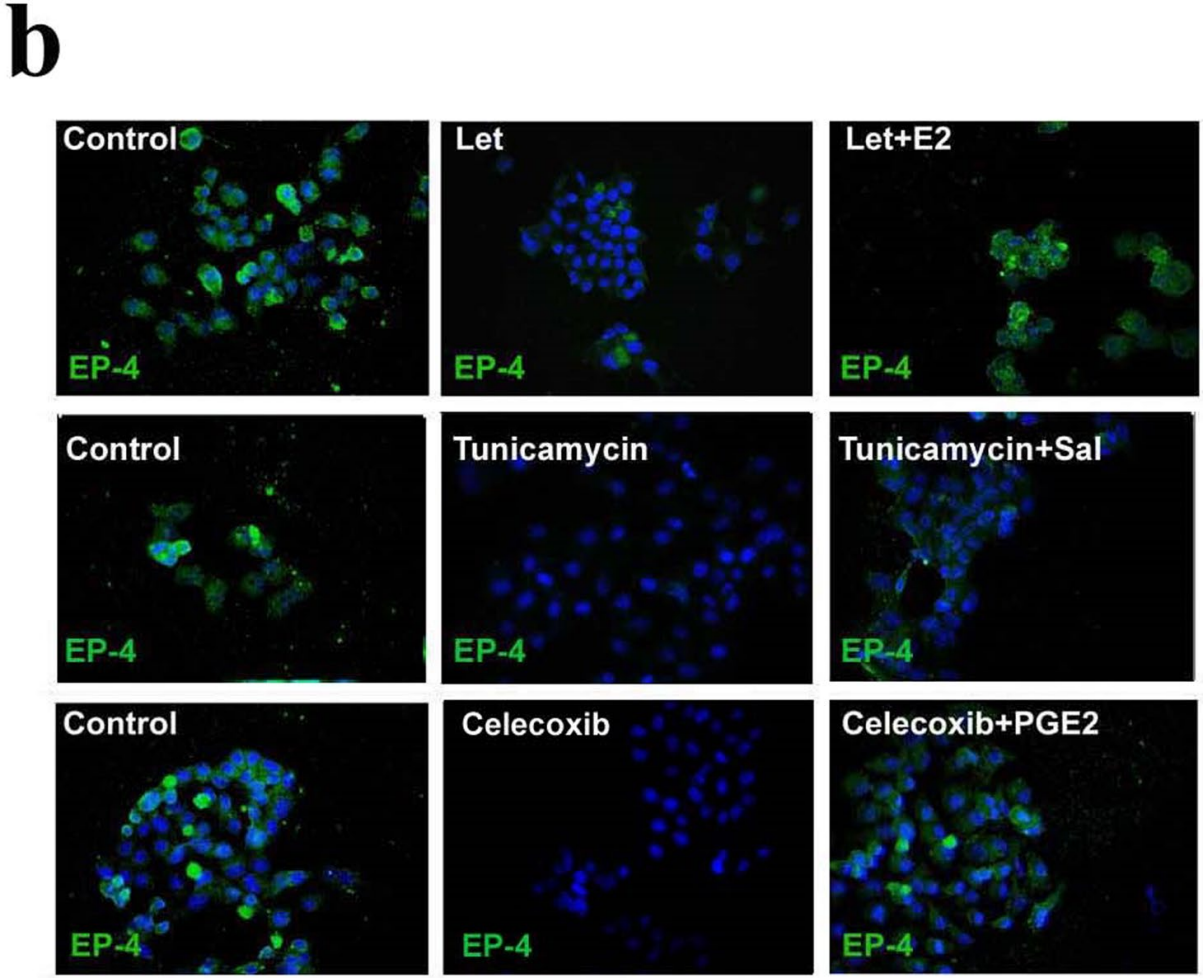
.

