# Supplementary material for: Menopause-induced uterine epithelium atrophy results from arachidonic acid/prostaglandin E2 axis inhibition-mediated autophagic cell death
Source: Sci Rep. 2016 Aug 10;6:31408. doi: 10.1038/srep31408 (PMC4979008; doi:10.1038/srep31408)
Supplement: Supplementary Information [file srep31408-s1.pdf]

Supplementary Information for

Menopause-induced uterine epithelium atrophy results from arachidonic acid/ prostaglandin E2 axis inhibition-mediated autophagic cell death

Shengtao Zhou§, Linjie Zhao§, Tao Yi, Yuquan Wei, Xia Zhao*

Department of Gynecology and Obstetrics, Key Laboratory of Obstetrics & Gynecologic and Pediatric Diseases and Birth Defects of Ministry of Education, West China Second Hospital, and The State Key Laboratory of Biotherapy, West China Hospital, Sichuan University, Chengdu, 610041, P. R. China

*** To whom correspondence may be addressed:** Xia Zhao, Department of Gynecology and Obstetrics, West China Second Hospital, Sichuan University, Chengdu, 610041, People's Republic of China, Tel: +86-28-85501633, Fax: +86-28-85164046, E-mail: xia-zhao@126.com

§ These authors contributed equally to this work.

Supplementary Figure 1

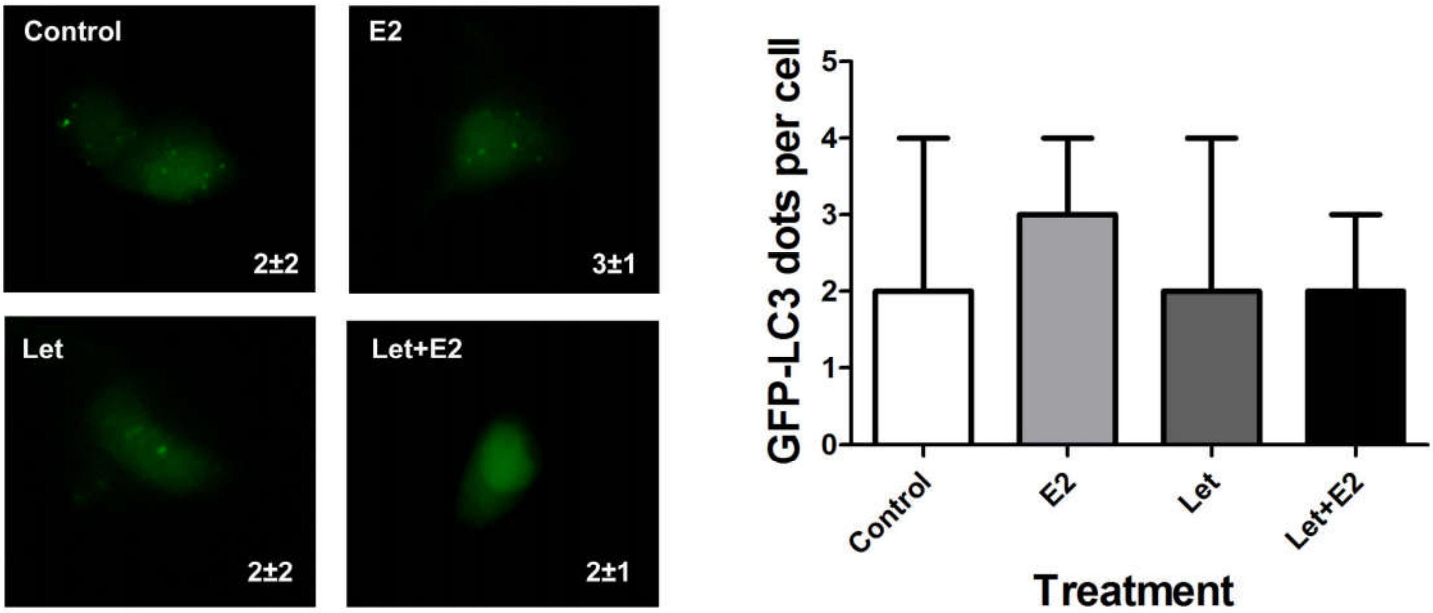

Supplementary Figure 1. Effects of estrogen deprivation on primary human endometrial stromal cells. Human primary endometrial stromal cells treated with DMSO (control), E2, letrozole or letrozole +E2 for 48 h were analyzed for LC3 using immunofluorescent analysis.

Supplementary Figure 2

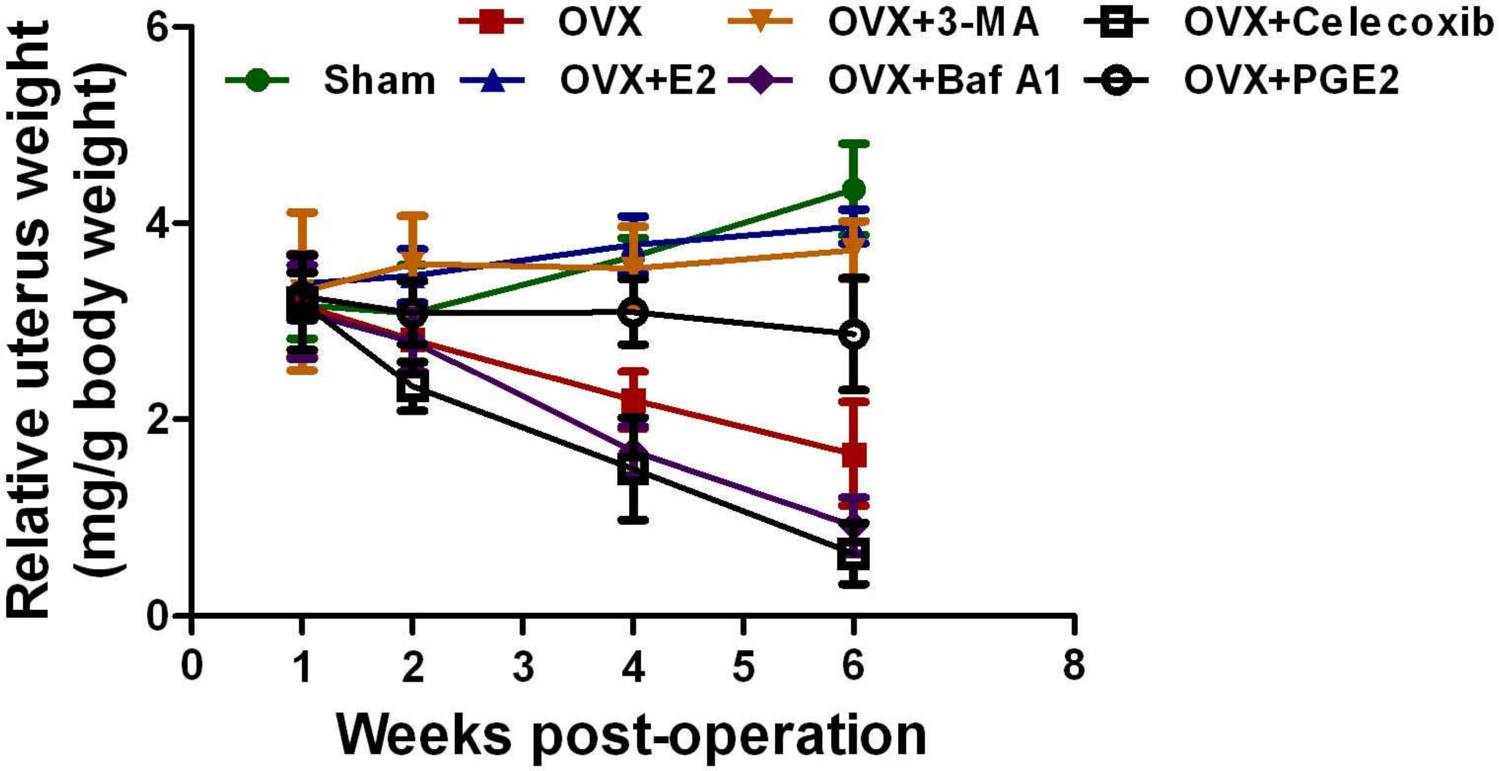

Supplementary Figure 2. Effects of manipulation of estrogen level, AA/PGE2 axis activity and autophagy level on relative uterus weight in ovariectomized rats. Relative uterus weight curves of female rats in sham-operated rats and ovariectomized rats treated with E2, 3-MA, Baf A1, celecoxib and PGE2.

Supplementary Figure 3

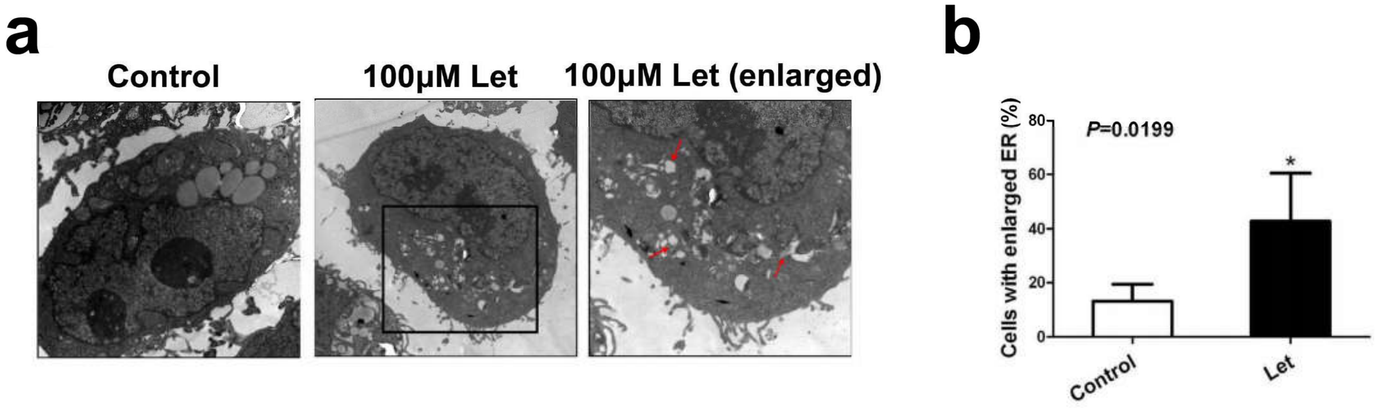

Supplementary Figure 3. TEM analysis of EECs treated with letrozole. (a) Representative TEM photograghs of EECs treated with letrozole. Note the presence of the dilated ER in letrozole-treated EECs. Red arrows point to the ER. (b) Quantification of the percentage of cells with enlarged ER relative to the total cell number.

Supplementary Figure 4

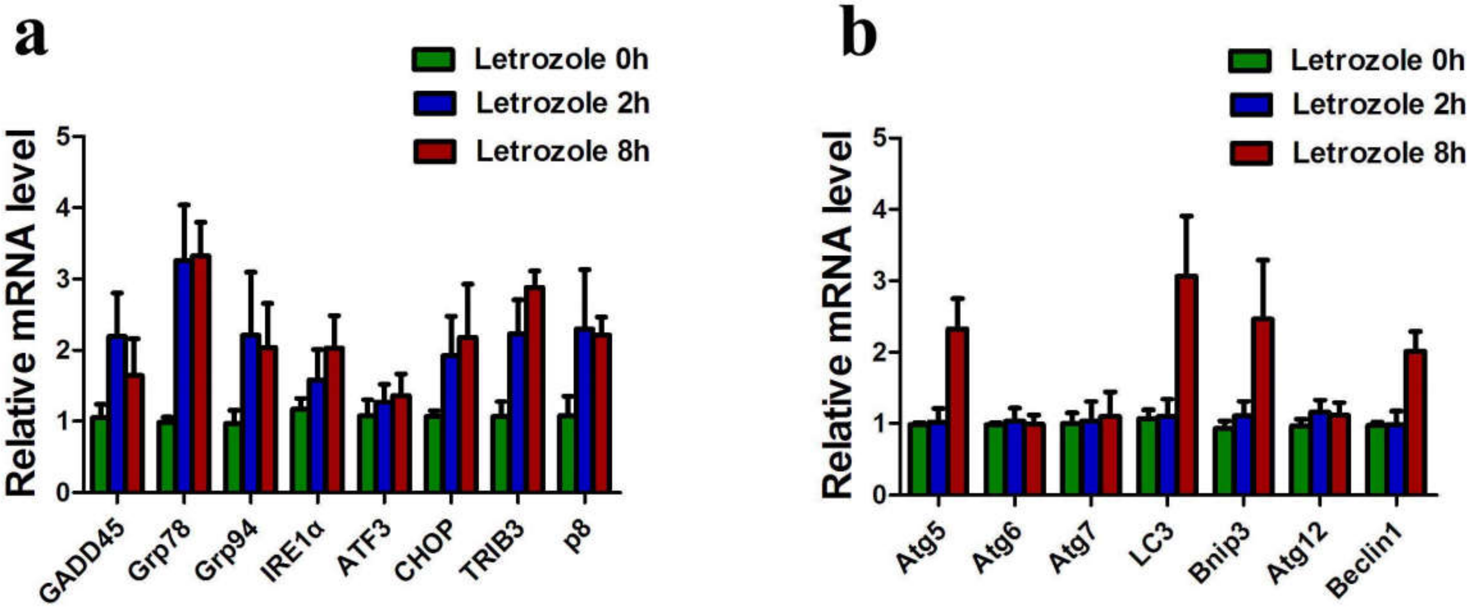

Supplementary Figure 4. Upregulation of ER stress genes occurred prior to autophagy gene upregulation in letrozole-treated primary endometrial epithelial cells. (a) Semi-quantitative PCR analysis was performed in triplicates using specific oligonucleotides for ER stress genes (see Supplementary Table 4) in letrozole-treated ECCs at indicated time points. (b) Semi-quantitative PCR analysis was performed in triplicates using specific oligonucleotides for autophagy genes (see Supplementary Table 3) in letrozole-treated EECs at indicated time points.

Supplementary Figure 5

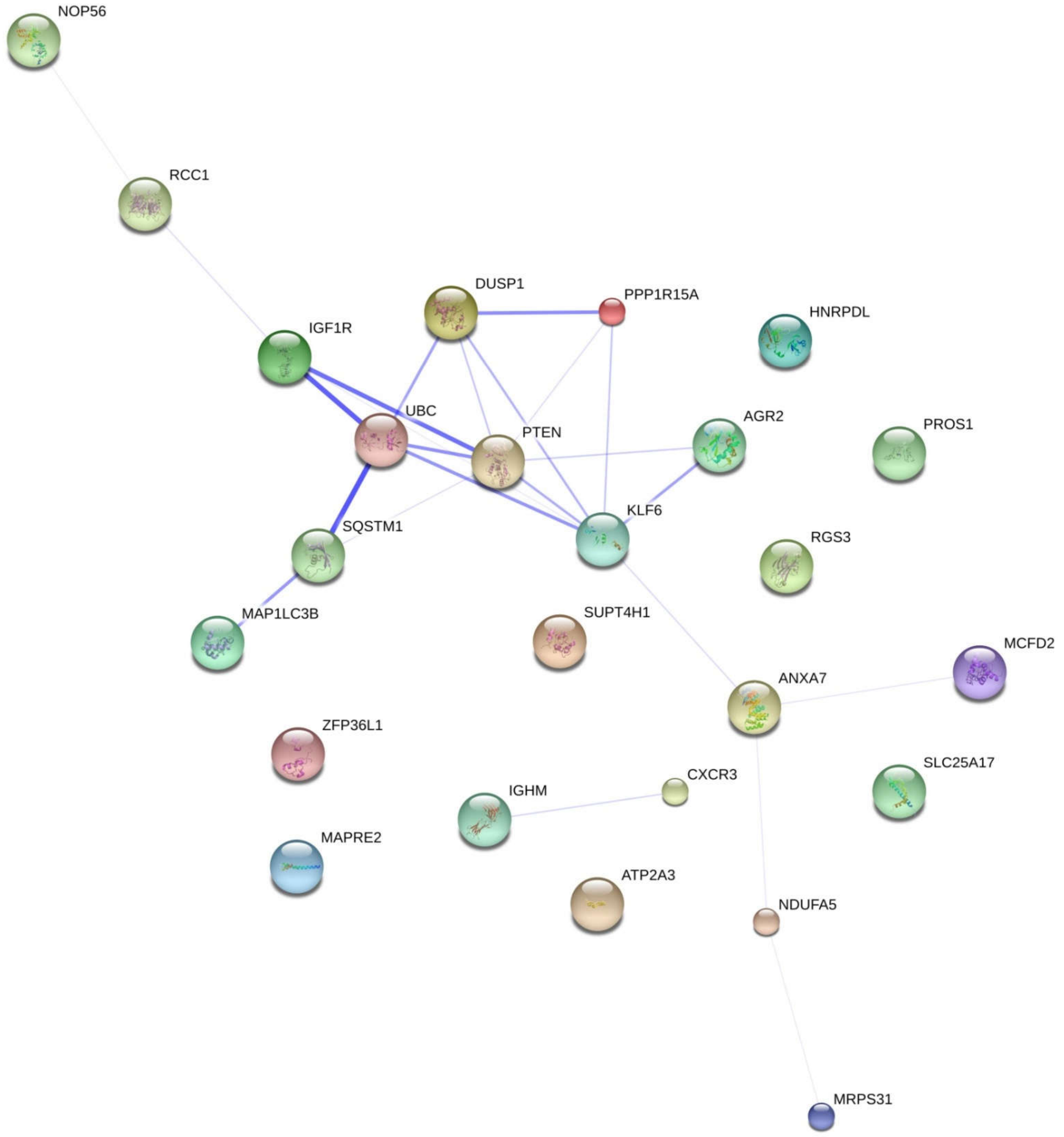

Supplementary Figure 5. The ovariectomy-induced ER stress network map. A STRING map of protein interactions in 25 commonly altered genes in ovariectomized mouse uteri and ER stress-challenged cells.

Supplementary Figure 6

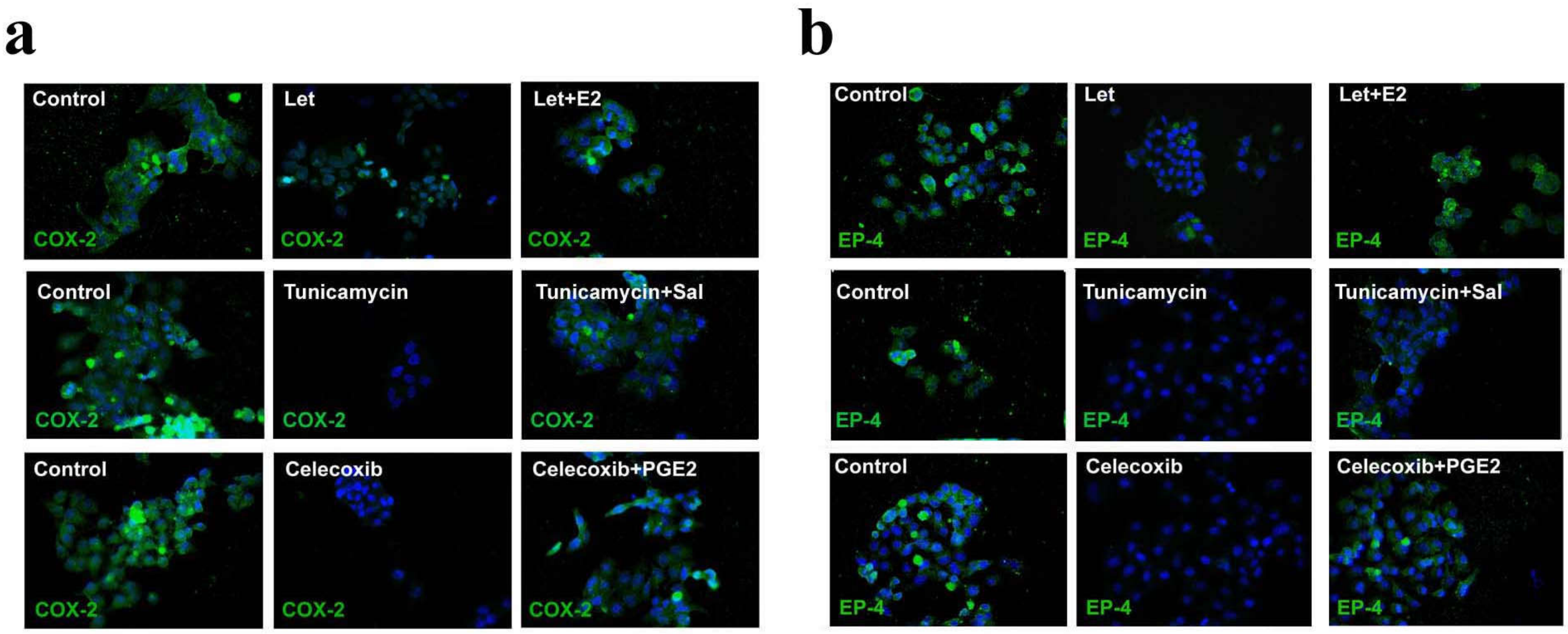

Supplementary Figure 6. Estrogen depletion-induced ER stress suppresses AA/PGE2 axis. (a) Immunofluorescent analysis of COX-2 expression (in green) in letrozole-treated and tunicamycin-treated EECs. Celecoxib treatment group was used as positive control. (b) Immunofluorescent analysis of EP-4 expression (in green) in letrozole-treated and tunicamycin-treated EECs. Celecoxib treatment group was used as positive control.

Supplementary Figure 7

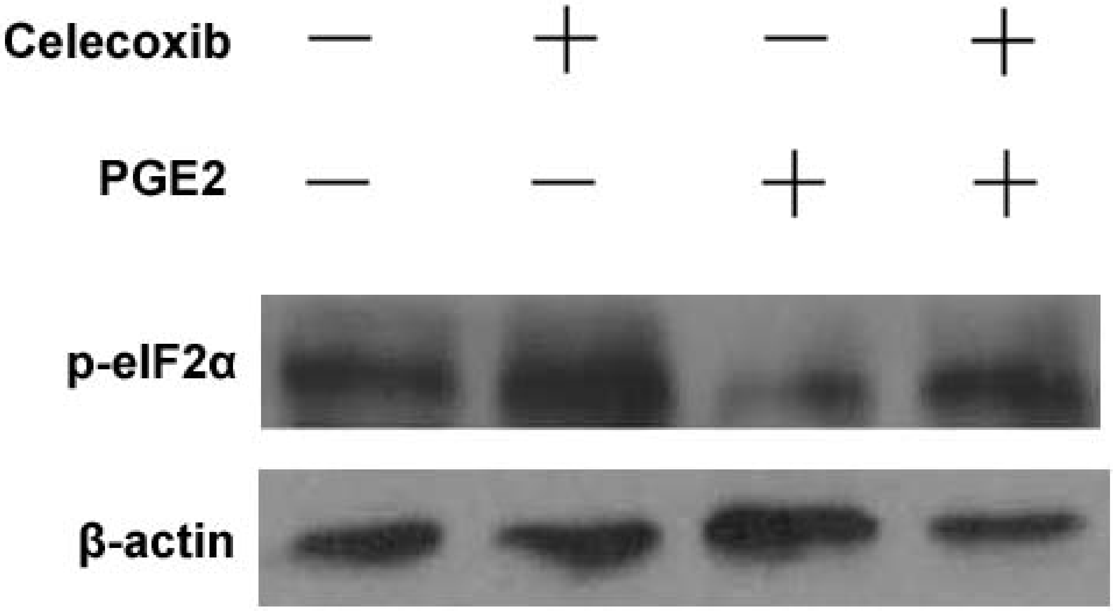

Supplementary Figure 7. The effect of AA/PGE2 axis on ER stress. Western blotting of p-eIF2α levels in EECs by exposure to celecoxib and PGE2.

Supplementary Figure 8

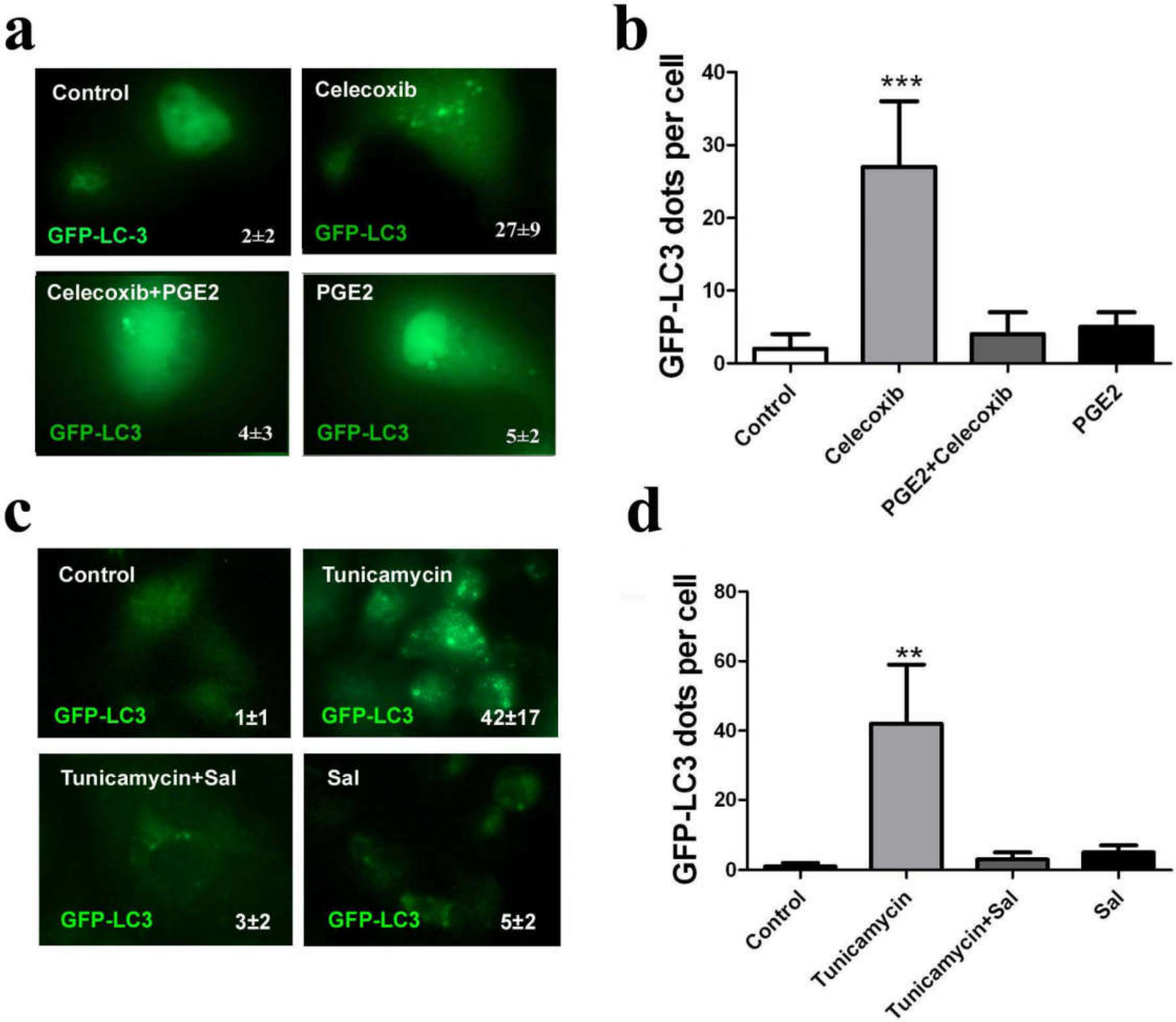

Supplementary Figure 8. ER stress-induced AA/PGE2 axis inhibition triggers autophagy induction. (a) EECs transfected with a GFP-LC3 plasmid were treated with DMSO (control), celecoxib, celecoxib+PGE2 or PGE2 and cells with GFP-LC3 punctate dots were examined at 24 h. (b) Quantification of GFP-LC3 dots in EECs treated with DMSO (control), celecoxib, celecoxib+PGE2 or PGE2 from supplementary Fig. 5a. (C) EECs transfected with a GFP-LC3 plasmid were treated with DMSO (control), tunicamycin, tunicamycin+salubrinal or salubrinal and cells with GFP-LC3 punctate dots were examined at 24 h. (D)

Quantification of GFP-LC3 dots in EECs treated with DMSO (control), tunicamycin, tunicamycin+salubrinal or salubrinal from supplementary Fig. 5a.

Supplementary Table 1. Publicly available microarray datasets of either ovariectomy or ER stress				
**Dataset No.**	**Treatment**	**No. of differential genes (P<0.05)**	**Common genes in three datasets**	**Reference**
GSE4664	Uterus treated by E2 in ovariectomized mice	4974	25	Hewitt et al (2003)
GSE2082	Mouse embryonic fibroblasts treated with tunicamycin	1578		Marciniak et al (2004)
GSE2883	Human cerebral vascular smooth muscle cells treated with thapsigargin	1269		Pulver-Kaste et al (2006)

Supplementary Table 2. List of Identified Estrogen-responsive Genes in Ovariectomized Uterus(GSE4664)			
**Gene ID**	**Gene Name**	**Log2 fold change**	**P value**
A_51_P402458	ANXA7	-3.22	0.0122
A_51_P505998	ATP2A3	-3.41	0.0358
A_51_P235798	ZFP36L1	-3.94	0.0062
A_51_P438155	MAPRE2	-6.04	0.0026
A_51_P451346	KLF6	-3.15	0.0003
A_51_P430900	DUSP1	-99.85	0.045
A_51_P319022	CXCR3	-2.45	0.0019
A_51_P318366	IGF1R	-12.97	<0.0001
A_51_P461902	IGHM	-3.36	0.0225
A_51_P302503	PPP1R15A	-5	0.0331
A_51_P393426	PROS1	-12.53	0.0001
A_51_P126181	MAP1LC3B	-2.51	0.0002
A_51_P211980	RGS3	-3.92	0.0057
A_51_P454300	SUPT4H1	-9.23	0.0004
A_51_P292097	UBC	-8.2	0.008
A_51_P119077	SQSTM1	-17.4	0.0001
A_51_P476091	HNRPDL	8.65	<0.0001
A_51_P319732	MRPS31	4.55	0.0324
A_51_P181170	SLC25A17	2.43	0.0086
A_51_P187901	NOP56	2.6	0.0495
A_51_P209122	AGR2	2.12	0.016
A_51_P465232	MCFD2	34.33	0.0027
A_51_P199927	RCC1	6.9	0.001
A_51_P170156	NDUFA5	2.56	0.0049
A_51_P275350	PTEN	1.75	0.0027

Supplementary Table 3. Primers for autophagy genes	
**Gene Name**	**Sequence**
Atg5	Sense 5′-GACAAAGATGTGCTTCGAGATGTG-3′
	Antisense 5′-GTAGCTCAGATGCTCGCTCAG-3′
Atg7	Sense 5′-ATGCCAGGACACCCTGTGAACTTC-3′
	Antisense 5′-ACATCATTGCAGAAGTAGCAGCCA-3′
LC3b	Sense 5′-CGGAGCTTTGAACAAAGAGTG-3′
	Antisense 5′-TCTCTCACTCTCGTACACTTC-3′
Bnip3	Sense 5′-CCACCTCGCTCGCAGACACCAC-3′
	Antisense 5′-GAGAGCAGCAGAGATGGAAGGAAAAC-3′
Atg12	Sense 5′-CCTCGGAACAGTTGTTTATT-3′
	Antisense 5′-CAGGACCAGTTTACCATCAC-3′
Beclin1	Sense 5′-CTGAAACTGGACACGAGCTTCAAG-3′
	Antisense 5′-CCAGAACAGTATAACGGCAACTCC-3′

Supplementary Table 4. Primers of ER stress genes	
**Gene Name**	**Sequence**
GADD45	Sense 5′-ATGACTTTGGAGGAATTCTCG-3′
	Antisense 5′-CACTGATCCATGTAGCGACTT-3′
GRP78	Sense 5′-GATAATCAACCAACTGTTAC-3′
	Antisense 5′-GTATCCTCTTCACCAGTTGG-3′
GRP94	Sense 5′-CAGTTTTGGATCTTGCTGTGG-3′
	Antisense 5′-CAGCTGTAGATTCCTTTGC-3′
IRE1α	Sense 5'-CCCAAATGTGATCCGCTACT-3'
	Antisense 5'-TTGAGAGAATGCAGGTGTGC-3'
ATF3	Sense 5′-CCTCGGAAGTGAGTGCTTCT-3′
	Antisense 5′-ATGGCAAACCTCAGCTCTTC-3′
CHOP	Sense 5′-GCACCTCCCAGAGCCCTCACTCTCC-3′
	Antisense 5′-GTCTACTCCAAGCCTTCCCCCTGC-3′
TRIB3	Sense 5'-TGCCCTACAGGCACTGAGTA-3'
	Antisense 5′-GTCCGAGTGAAAAAGGCGTA-3′
p8	Sense 5′-GGCACGATGGCCACCTTCCCACC-3′
	Antisense 5′-CTCATCTCCAGCTCTGTCTCAGCG-3′

Supplementary Table 5. Target sequences of each siRNA	
**Gene Name**	**Sequence**
Atg5	5'-CAACTTGTTTCACGCTATA-3'
Beclin 1	5'-GGACAGUUUGGCACAAUCA-3'

Supplementary Table 6: Clinicopathological data of patients from whom the samples for primary culture experiments were collected.		
**Patient No.**	**Age**	**Sampling time during menstrual cycle**
1	36	Proliferative
2	41	Proliferative
3	31	Secretory
4	25	Proliferative
5	28	Secretory
6	42	Proliferative
7	33	Proliferative
8	43	Secretory
9	29	Proliferative
10	37	Secretory
